# Vaginal Aging—What We Know and What We Do Not Know

**DOI:** 10.3390/ijerph18094935

**Published:** 2021-05-06

**Authors:** Jacek K. Szymański, Aneta Słabuszewska-Jóźwiak, Grzegorz Jakiel

**Affiliations:** First Department of Obstetrics and Gynecology, Centre of Postgraduate Medical Education, Żelazna 90 Str., 01-004 Warsaw, Poland; anetaslabuszewska@gmail.com (A.S.-J.); grzegorz.jakiel1@o2.pl (G.J.)

**Keywords:** menopause, vaginal microbiota, estrogens, DNA methylation, TET protein

## Abstract

The aging of the organism is a complex and multifactorial process. It can be viewed in the context of the whole organism, but also of individual tissues and organs. The problem of vaginal aging and the related genitourinary syndrome of menopause significantly reduces the quality of women’s lives. The aging process of the vagina includes estrogen deficiencies, changes in the microbiome, and changes at the genetic level associated with DNA methylation. During the menopause, the number of Lactobacillus colonies decreases, and the number of pathological bacteria colonies increases. The decrease in estrogen levels results in a decrease in vaginal epithelial permeability, perfusion, and elastin levels, resulting in vaginal dryness and atrophy. Changes at the molecular level are the least clear. It can also be assumed that, similarly to the tissues studied so far, there are changes in cytosine methylation and TET (ten-eleven translocation) expression. The interrelationships between DNA methylation, hormonal changes, and the vaginal microbiome have not yet been fully elucidated.

## 1. Introduction

**Figure 1:**
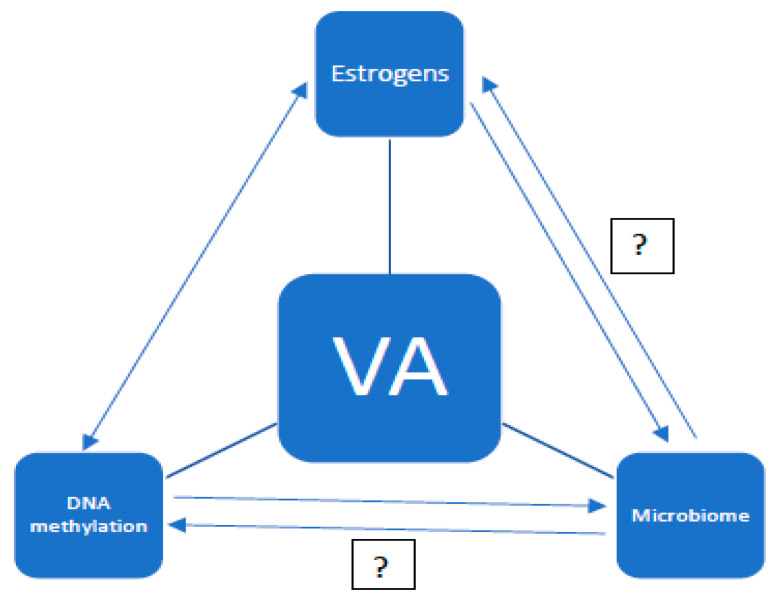
Potential relationships between estrogen levels, the vaginal microbiome, and DNA methylation in vaginal aging (VA—vaginal aging).

The aging of the organism is an extremely complex and multifactorial process. It consists of changes at the genetic, hormonal, and immunological levels. Moreover, the problem of aging can be considered in terms of the whole organism, individual organs, and tissues. The aim of this study is to present the current knowledge on the aging of the vagina. So far, the vaginal microbiota and the effects of estrogens on the vaginal epithelium, as well as the effects of estrogen deficiency, have been well understood. Undoubtedly, the vaginal microbiome is dependent on estrogen levels. However, there are no studies on the genetic aspects of the aging of the vaginal mucosa. It has been proven that DNA (deoxyribonucleic acid) methylation is associated with the aging process [1,2]. However, it is unclear whether DNA methylation is the cause of aging, an effect of it, or just a concomitant process. The analysis of DNA methylation in other tissues confirms the tissue specificity of this process. It can be presumed that the processes of DNA methylation and demethylation in the vagina will also have a specific course, and that the molecular processes of vaginal aging involve the cycle of dependence with the influence of estrogens and the microbiome. However, certain relationships have not yet been clarified (see Figure 1).

## 2. Changes in the Field of Microbiota

From the beginning of the existence of the human species, various bacterial colonies, generally known as the microbiota, inhabited different locations of the human body, and evolved along with the evolution of man. This co-evolution, as well as the transmission of microbes within a species over successive generations, has been proven on the basis of the convergence of the phylogenetic tree of microbiota and primates [3]. At the same time, an immune tolerance was developed, aimed at the elimination of invasive microbes and the protection of its own bacteria [4]. Socioeconomic development has caused changes in lifestyle, diet, and hygiene habits that, together with the introduction of antibiotics, has resulted in significant changes in the human microbiome. Another significant factor is the increase in deliveries by caesarean section in recent years, which disrupts the transmission of normal bacterial flora from the mother to the newborn. A microbiota disorder is currently believed to be the basis of many autoimmune diseases, allergies, obesity, and type II diabetes [5,6]. The gut microbiome has been best understood thus far. Changes in the intestinal microflora accompanying the aging of the organism have been observed. Moreover, a specific type of gut microbiota has been found in centenarians [7,8,9]. Another well-known microbiome is the vaginal flora dominated by the Lactobacillus species [10]. The Lactobacillus species are involved in the production of lactic acid and hydrogen peroxide, which ensure the right pH in the vagina, thus contributing to the protection of the vaginal environment against invasive bacterial strains [11]. A reduction in the number of Lactobacillus sp. often results in infection with anaerobic bacteria, leading to bacterial vaginosis and, in pregnant women, to preterm labor. The bacterial flora of the vagina dynamically changes under the influence of many factors, including lifestyle, smoking, hygiene habits, sex life, menstrual cycle, hormonal contraceptives, HPV (Human Papillomavirus) infections, Trichomonas vaginalis, and Chlamydia trachomatis, but mainly due to changes in the hormonal status of women resulting from age and the aging process [12,13,14,15,16,17,18,19]. The first significant changes in the bacterial environment of the vagina occur during puberty. Early puberty is characterized by a low Lactobacillus vaginal flora with the dominance of bacterial strains of intestinal origin. About 1 year before the menarche, the vaginal bacterial environment begins to resemble the microbiome of Lactobacillus-dominant women in their reproductive years [20]. The period of pregnancy is particularly important for the maintenance of normal bacterial flora, as the relationship between microbiota disorders and preterm labor has been proven [21,22]. During the menopause, a marked decrease in the number of Lactobacillus and an increase in colonies of pathological bacteria is observed, especially in women complaining of GSM (genitourinary syndrome of menopause) [11,23]. One study using the community state types (CSTs) scale showed marked differences in vaginal microbiomes between premenopausal, perimenopausal, and postmenopausal women. In women of reproductive age, CST-I or CST-III dominated; in perimenopausal CST-IVA, Lactobacillus gasseri dominated, while postmenopausal women were classified as CST-IVA/IVB or CSTV [13]. There was also a clear relationship between the bacterial state of the CST-IVA vagina and vulvovaginal atrophy [24]. What is more, hormone replacement therapy reducing GSM symptoms has a positive effect on the vaginal microbiome, restoring the dominance of Lactobacillus [25,26]. It can therefore be hypothesized that bacterial flora, dominated by Lactobacillus in postmenopausal women, improves quality of life.

## 3. The Influence of Hormones on the Vaginal Epithelium

**Figure 2:**
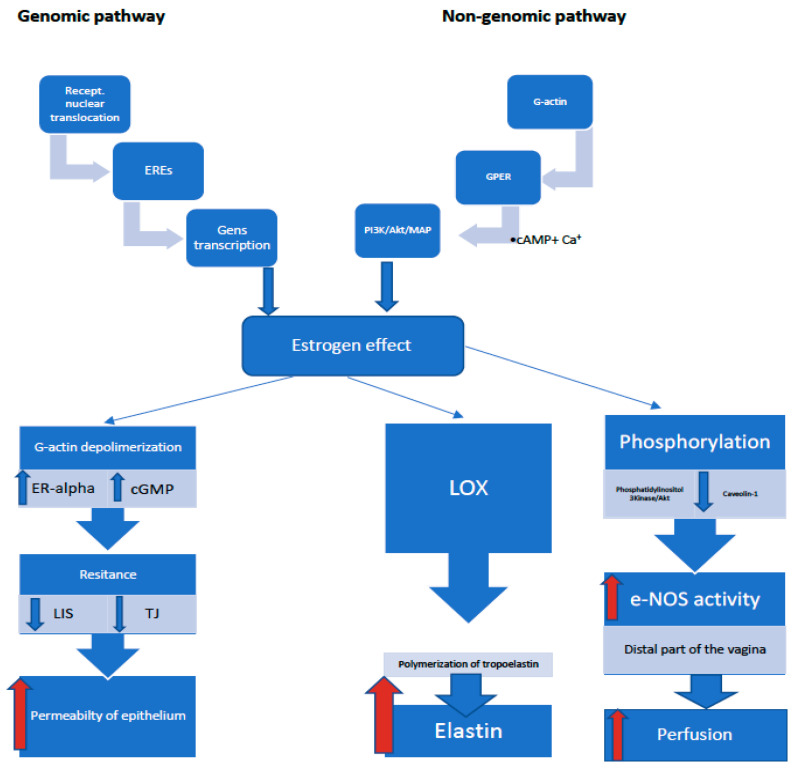
The mechanism of action of estrogens. EREs—Estrogen response elements; GPER—G-protein-coupled estrogen receptor; PI3K/Akt—Phosphatidylinositol 3Kinase/Akt; ER-alpha—Estrogen receptor alpha; cGMP—Cyclic guanosine monophosphate; LIS—Lateral intracellular space; TJ—Tight junctions, LOX—Lysyl oxidase. The estrogen effect is induced in two ways: through genomic and non-genomic pathways. In genomic pathways, the induction of receptor nuclear translocation leads to an attachment to the EREs, with the subsequent regulation of the transcription of the target genes. In non-genomic pathways, GRER is activated, involving G-actin, with the subsequent induction of cytoplasmic pathways PI3K/Akt/MAPK/ERK ½ and p38 via cAMP activation and the mobilization of intracellular calcium. Subsequently, three parallel processes are involved in obtaining the estrogenic effect in the vaginal epithelium. The stimulation of ER-alpha is accompanied by the activation of cGMP, causing the depolymerization of G-actin with a direct impact on the reduction in LIS and TJ. The phosphorylation of eNOS and the inhibition of the interaction of the caveolin-1 protein increase eNOS activity, thus decreasing LIS and TJ resistance. These two processes result in the increased permeability and perfusion of the epithelium and, subsequently, reduced vaginal dryness. Moreover, estrogens affect the expression of LOX, which catalyze the polymerization of tropoelastin monomers, increasing the level of elastin in the vagina, thus restoring vaginal elasticity.

The effect of steroid hormones on the vaginal mucosa is indisputable and proven. The mature four-layer epithelium characteristic of a woman’s reproductive period begins to atrophy as estrogen levels decline during the menopause. This aging atrophy results in the thinning of the epithelium, the smoothing of mucous folds, reduced blood flow, pallor, and greater trauma, as well as decreased epithelial glycogen content, reduced lubrication, and dryness. These changes also apply to the stroma. They are characterized by an increase in the collagen and lipofuscin content, as well as an influx of plasma cells and lymphocytes. The vagina is the target organ for estrogen. Estrogen receptors (ER) are found in the basal and parabasal epithelial layer, in the stroma, and in the smooth muscle of pre- and post-menopausal women. Thus, the topical application of estrogens should affect both vaginal epithelial cells and connective tissue, especially collagen synthesis [27]. The topical application of estrogens restores the layering of the epithelium, the elasticity of the vagina, lowers the pH and, as mentioned above, affects the quality of the vaginal microbiome. There are reports that the vaginal administration of estrogens is limited to the epithelium only, without affecting the extracellular matrix and neovascularization [28]. Estrogens exert a biological effect by activating the estrogen receptors, ER-alpha and ER-beta, the distribution of which in the vagina depends on the woman’s menopausal status. The expression of ER-alpha receptors is observed before and after the menopause, while ER-beta receptors are found in women of reproductive age [29]. Immunohistochemical studies have shown the differential expression of ERs in vaginal tissue. ER-alpha receptors were found in the vaginal epithelial and the stromal and smooth muscle cells, while ER-beta receptors were detected in the epithelial and vascular smooth muscle cells of the vagina. In turn, ER-alpha were not found in the vaginal blood vessels. It is therefore likely that the decrease in ER-beta expression after the menopause is associated with an unfavorable vascular effect in the vaginal epithelium. Thus, while both types of estrogen receptors play important roles in the vaginal wall, the expression of ER-alpha is not correlated with that of ER-beta [30,31,32]. After the menopause, the expression of ER-beta receptors is significantly reduced, regardless of estrogen therapy. Estrogen receptor stimulation occurs in two ways. The classic way is based on receptor nuclear translocation induction and attachment to estrogen response elements (EREs), with subsequent regulation of the transcription of target genes by the direct effect of Akt on DNA. In this way, mitochondrial functions, such as mitochondrial biogenesis (NRF-1 stimulation) and DNA transcription, oxygen consumption and apoptosis, are controlled. Thus, impaired mitochondrial function in the case of an estrogen deficiency may accelerate the aging process. The non-classical, non-genomic pathway involves the activation of a membrane-associated receptor, such as the G-protein-coupled estrogen receptor (GPER) and the induction of cytoplasmic pathways, such as the PI3K/Akt/MAPK/ERK ½, and p38, through cAMP (adenosine cyclic monophosphate) activation and intracellular calcium mobilization, leading to an immediate estrogenic effect. Moreover, the stimulation of estrogen receptors activates growth factors, such as EGFR, HER2/Neu, and IGFR [33,34,35,36,37,38] (see Figure 2). The alkalization of the vaginal environment, along with a decrease in the concentration of estrogens and a reduction in Lactobacillus bacteria are observed in the process of vaginal aging. The acidification of the vaginal environment process is mediated by the active secretion of H+ by V-H+ -ATPase in the cell membrane. A study by Gorodeski showed that topical estrogen therapy in postmenopausal women restores the acidic pH of the vagina only to a certain extent and does not lead to obtaining the values of premenopausal women. Thus, aging-related hypoestrogenism is probably not the only factor contributing to the maintenance of an acidic vaginal environment, but there are other still unknown factors that influence this process [39]. Vaginal dryness is another symptom of aging. Dryness directly relates to the permeability of the vaginal epithelium and is dependent on paracellular permeability, determined by the resistance of intercellular tight junctions (TJ) and lateral intercellular space (LIS). Tight junction areas close intercellular spaces, generating a high resistance to epithelial permeability, while intercellular spaces are considered to be low-resistance areas. Subsequent studies by Gorodeski showed an increase in resistance associated with age and ageing, especially within the LIS. It is probable that the increase in LIS resistance is influenced by the reduced capillary pressure in the vaginal mucosa as a result of aging. On the other hand, the effect of hypoestrogenism itself was shown to increase the resistance within TJ, regardless of the aging process. These changes result in a decrease in the permeability of the vaginal epithelium, leading to a disturbance of plasma transport from the blood to the vaginal lumen and subsequent dryness [40,41]. Furthermore, an aging-related reduction in cellular G-actin, a component of the cytoskeleton involved in regulating the cell’s life cycle, has also been demonstrated. Aging causes a decrease in G-actin levels, leading to a shift from the actin steady state to polymerization and the formation of a rigid cytoskeleton, resulting in a decrease in epithelial permeability. In turn, estrogen therapy by the depolymerization of actin reduces LIS resistance by increasing the permeability of the epithelium. The increase in G-actin levels after estrogen therapy results from the activation of the ER-alpha receptor, as well as the nitric oxide (NO)-related activation of cGMP. What is more, estrogens improve gap-junctional intracellular communication (GJIC); hence, the decreased level of estrogens will affect the regulation of intercellular communication, thus accelerating the aging process [38]. It is highly probable that NO interaction is involved in the aging process. The relationship between estrogen levels and epithelial NO synthase (eNOS) activity is not fully understood. Endothelial NO is known to affect vasodilation and improve perfusion in the vaginal epithelium. Rodent studies suggest species specificity regarding the effects of estrogens on eNOS. However, it seems more important to differentiate the effect of estrogens on the activity of eNOS and arginase depending on the vaginal region. In studies on rabbits, it was shown that an ovariectomy increased, while estrogens decreased, the activity of NOS and the expression of the eNOS protein in the proximal part of the vagina. On the contrary, in the distal part, an increase in the activity of eNOS and arginase was observed under the influence of estrogens, and a decrease in activity was found after an ovariectomy [42]. The post-translational activation of eNOS by estrogens occurs by two mechanisms. The first is related to the phosphorylation of eNOS on Ser-1177, via phosphatidylinositol 3-kinase/Akt, and the second to the inhibition of interaction with its negative regulator caveolin-1 protein. The lack of estrogens decreases phospho-eNOS levels in vaginal tissue and increases eNOS binding to caveolin-1. In turn, the inhibition of eNOS activity after binding to caveolin-1 is caused by Ser-114 phosphorylation, which normalizes under the influence of estrogen therapy [42]. At reproductive age, the level of estrogen is sufficient to maintain low TJ and LIS resistance and, therefore, high epithelial permeability. Estrogen therapy in postmenopausal women primarily decreases LIS resistance, with therapeutic efficacy improving with the duration of the therapy, but in a manner inversely proportional to age. Prolonging estrogen therapy may also lower TJ resistance by increasing the permeability of the vaginal epithelium [41]. Moreover, there is a likelihood of a beneficial effect of estrogens on the expression of lysyl oxidase (LOX) and LOX-like proteins, which catalyze the polymerization of tropoelastin monomers to elastin. Therefore, it can be assumed that one of the causes of vaginal atrophy may be an estrogen deficiency resulting from the aging process, leading to a decrease in the expression of the LOX family proteins and a decrease in the level of elastin in the vagina [43] (see Figure 2). Estrogens are an important regulator of DNA methylation. Estrogen signaling is associated with changes in DNA methylation in breast cancer cells, as well as the regulation of epigenetic enzymes in the hypothalamus. It has also been shown that, through methylation, estrogens inhibit the expression of vascular soluble epoxide hydrolase, thus increasing the level of epoxyeicosatrienoic acids, which improve tissue perfusion through vasodilatation [44]. The stabilizing effect of estrogens on intracellular proteins, the accumulation of which may lead to apoptosis and accelerated aging, also seems to be important [45].

## 4. Genetic Aspects of Aging

It is known that the ageing process of the organism is influenced by epigenetic changes, such as DNA methylation, the modification of histone proteins, or the expression of non-coding RNA (ribonucleic acid). While aging is associated with disturbances in DNA methylation, it is unclear whether it is the changes in DNA methylation alone that contribute to the aging process. In general, global hypomethylation and local hypermethylation are observed in the aging process [1]. The methylation of cytosine in CpG (5′-C-phosphate-G-3′) pairs, densely packed around the promoter, inactivates the gene, while methylation of rare CpG pairs causes the activation of the gene. In turn, the demethylation of cytosine in DNA usually activates genes [46]. In a study by Borkowska and colleagues, an increase in 5 mC (5 methylcytosine) in subcutaneous adipose tissue was shown, and ASCs (Adipose Stem Cells) correlated with the aging of the organism, with simultaneous hypomethylation in differentially hydroxymethylated regions (DHMRs). There was no correlation between the global 5 mC level and the expression of any of the TET (ten-eleven translocation) genes, although an age-related increase in TET2 mRNA and TET3 proteins was observed. It is possible that the ability of TET to convert 5 mC to 5fC (5 fluorocytosine) decreases with age, leading to the accumulation of 5 mC [2]. Similarly, an increase in 5 mC was observed in senescent human T cells. A reduced expression of TET 1 and TET3 mRNA was also noted, as well as no change in the expression of TET2 mRNA. There was also a correlation between TET3 expression and 5 mC content, with no such correlation in TET 1 and TET2 [47].

In the aging process, there is a global decline in DNA methylation. At the same time, the overall content of the epigenetic marker 5 mC increases with age, with no increase in TET 1-3 mRNA expression [48]. In mammals, DNA methylation is mainly located in CpG dinucleotides. The distribution of the CpG varies. High-density CpGs, the so-called CpG islands (CGIs), most often unmethylated, are located within the promoter regions. Conversely, the regions of low CpG density located primarily in repetitive DNA are heavily methylated. The third group comprises the low methylated regions (LMRs), dependent on the transcription factor (TF) binding site [1].

DNA methylation plays an essential role in the regulation of gene expression in mammals. The methylation process is tissue- and cell-specific through the action of cytosine DNA methyltransferases (in mammals: DNMT1, DNMT3A, and DNMT3B) [1]. 5 mC, also known as the sixth base, plays a special role in the regulation of gene expression. The level of 5 mC in gene bodies positively correlates with gene expression [48]. With the aging of the organism, an increase or decrease in cytosine methylation is observed, depending on the type and region of DNA [47]. In the cerebellum, a genomic increase of 5 mC was noted, with no changes in its content in mitochondrial DNA. A similar increase in genomic 5 mC was observed in sperm [47]. The DNA methylation process generally silences genes, although the role of methylation in gene activation remains unclear. The influence of environmental factors on the methylation process and the related gene activation or silencing mechanism seems to be highly probable [1]. The correct DNA methylation/demethylation process regulating correct transcription in the cell requires an appropriate interaction of DNA methyltransferases and TET proteins [49]. DNA demethylation, opposing methylation, may be passive during cell division in the absence of methylation or active, replication-independent, with the participation of TET family methylcytosine dioxygenases (ten-eleven translocation) [50]. TET causes the oxidation of 5 mC, generated by cytosine methylation and catalyzed by DNA methyltransferases (DNMTs), to 5 mC, 5fC, and 5caC (5 carboxylcytosine) in DNA. Additionally, thymine DNA glycosylase (TDG) restores unmethylated cytosine through active demethylation. Active demethylation also includes the DNA repair mechanism involving the excision of methylated cytosine and its replacement with unmethylated cytosine—the base excision repair (BER) pathway [46,51]. Passive demethylation, or rather the prevention of passive DNA methylation, is related to the inability to recognize 5 mC by DNMT1. In this way, the TET family controls the 5 mC and 5 hmC (5 hydroxymethylcytosine) levels through active demethylation or the negative regulation of passive DNA methylation [48].

The family of ten-eleven translocation (TET) dioxygenases is made up of TET 1, TET2, and TET3. Proteins have a similar structure built by the amino-terminal CXXC zinc-finger domain and the carboxyl-terminal catalytic dioxygenase domain. The CXXC zinc-finger domain consists of approximately 60 amino acids and is only present in TET 1 and TET3. The TET2 CXXC domain is a separate gene called IDAX or CXXC4 that negatively regulates TET2 [48,52]. The catalytic domain is made up of a Cys-rich segment, a double-stranded beta helix (DSBH) domain, and a non-conserved low-complexity insert (NCLC). The CXXC zinc-finger domain is responsible for the attachment to unmethylated CpG dinucleotides in DNA. TET2, in turn, binds to CpG dinucleotides with a preference for 5 mC. The level of 5 hmC is in the range of 5–40% 5 mC, depending on the type of cell. Interestingly, DNMT3A and DNMT3B also have the ability to directly convert 5 mC to cytosine. Moreover, the TET family, apart from its direct influence on DNA, modifies histones, thus influencing their stability and interaction with other proteins. This process occurs by recruiting O-linked β-d-N-acetylglucosamine transferase (OGT) to chromatin by binding TET to VprBP. OGT is present in the cytoplasm and cell nuclei, catalyzes serine and threonine glycosylation, and regulates TET phosphorylation [48]. Three mechanisms of regulation of gene transcription have been described:

1. Chromatin remodeling regulated by histone GlcNAcylation;
2. Transcriptional activation induced by the GlcNAcylation of HCF1 (host cell factor 1) promoted by the interaction of TET2/3 with OGT;
3. Facilitation of the biding of transcriptional factor-like molecules to the promoter of associated genes by the OGT–TET3 complex [48].

By attaching to CpG-rich regions, TET inhibits DNA methyltransferase activity, while converting 5 mC to 5 hmC. TET catalytic activity requires the presence of three cofactors: 2-oxoglutarate, Fe(II), and ascorbic acid, which prevents the oxidation of Fe(II). Vitamin C, as a cofactor of TET enzymes, induces TET-dependent DNA demethylation [48]. TET gene expression varies from tissue to tissue. In general, TET1 and TET2 mRNA levels decrease while TET3 mRNA levels increase during cell differentiation. In addition, the regulation of TET proteins also takes place at a non-translational level through caspase-dependent degradation and calpains proteases. OGT, by the acetylation of TET3, can prevent the formation of TET3-catalyzed 5 mC. The TET family plays an essential role in the development and differentiation of stem cells, thus contributing to the development of the embryo at various stages. It is not yet clear how TETs are involved in gene regulation in different cell types [53].

Differentially methylated regions (DMRs) are believed to be involved in the regulation of gene transcription. The distribution of DMRs is tissue-specific [2]. Together with CpG, they create the so-called epigenetic clock, determining the degree of tissue aging and lifespan [1].

CpGs and hypermethylated DMRs are located primarily on the genes involved in cell differentiation and development, and particularly within bivalent chromatin domain promoters. This is the region where the Polycomb target genes are located that regulate the silencing gene and genes that control development. The TET1 complex with the Polycomb Repressive Complex2 (PRC2) participates in epigenetic plasticity in the process of cell differentiation. Cell differentiation is thus regulated by the interaction of DNA methylation/demethylation and histone methylation/demethylation [48]. So far, it is not clear whether methylation can be directly related to the degree of aging of tissues and whether methylation causes aging, or is the result of this process [1]. Recent reports link DNA methylation with an influence on the energy metabolism through the regulation of the C/EBP-beta transcription factor (CCAAT/enhancer-binding protein-beta). C/EBPs play a key role in regulating glucose and fat metabolism. Impaired demethylation of C/EBP-beta sites accelerates the aging process. C/EPB belongs to the superfamily of transcription factors—the basic leucine zipper (bZIP)—controlled by the mechanistic/mammalian target of the rapamycin complex 1 (mTORC1), considered to be one of the key regulators of lifespan and health. mTORC1, by regulating C/EBP expression, influences the activation of the metabolic processes that are characteristic of caloric restrictions, as well as the production of specific proteins, thus affecting life expectancy [1]. The associations between metabolic processes, postmenopausal hormonal changes in a woman’s life, and DNA methylation require clarification. The relationship between estrogen deficiency and DNA methylation seems to be of particular interest. Ulrich and colleagues have shown inverse associations between sex hormones in overweight postmenopausal women with low serum folate levels and DNA methylation. Interestingly, such correlation was not observed among women with high folate levels and those who used multivitamins. This negative association between DNA methylation and sex hormones was apparent in women with a lower level of methyl donor S-adenosylmethionine resulting from a lower one-carbon status. Thus, the authors formulated the hypothesis that one-carbon status may be an important factor in the relationship between sex hormones and DNA methylation. However, direct evidence linking hormonal status to the methylation level is lacking [54].

## 5. Conclusions

The complexity of the aging process makes it difficult to understand exactly; the molecular factors that underlie this process especially need to be elucidated. Therefore, well-designed vaginal studies are needed to determine the epigenetic aspects of aging. What is more, research that will reveal the interrelationships between estrogen therapy and the process of DNA methylation in the vaginal mucosa will be highly interesting. It is possible that, in the future, epigenetic therapy influencing DNA methylation will contribute to the prolongation of the life and function of various body tissues, including vaginal tissues, contributing to the improvement of quality of life in the aging period.

## Data Availability

Publicly available datasets were analyzed in this study. This data can be found here: https://pubmed.ncbi.nlm.nih.gov.
